# Lowe syndrome

**DOI:** 10.1186/1750-1172-1-16

**Published:** 2006-05-18

**Authors:** Mario Loi

**Affiliations:** 1 Division of Paediatric Neurology, G. Brotzu Hospital, Cagliari, Italy

## Abstract

Lowe syndrome (the oculocerebrorenal syndrome of Lowe, OCRL) is a multisystem disorder characterised by anomalies affecting the eye, the nervous system and the kidney. It is a uncommon, panethnic, X-linked disease, with estimated prevalence in the general population of approximately 1 in 500,000. Bilateral cataract and severe hypotonia are present at birth. In the subsequent weeks or months, a proximal renal tubulopathy (Fanconi-type) becomes evident and the ocular picture may be complicated by glaucoma and cheloids. Psychomotor retardation is evident in childhood, while behavioural problems prevail and renal complications arise in adolescence. The mutation of the gene *OCRL1 *localized at Xq26.1, coding for the enzyme phosphatidylinositol (4,5) bisphosphate 5 phosphatase, PtdIns (4,5)P2, in the trans-Golgi network is responsible for the disease. Both enzymatic and molecular testing are available for confirmation of the diagnosis and for prenatal detection of the disease. The treatment includes: cataract extraction, glaucoma control, physical and speech therapy, use of drugs to address behavioural problems, and correction of the tubular acidosis and the bone disease with the use of bicarbonate, phosphate, potassium and water. Life span rarely exceeds 40 years.

## Disease name

Lowe and colleagues in 1952 described a unique syndrome with organic aciduria, decreased renal ammonia production, hydrophthalmos and mental retardation [1]. In 1954, a renal Fanconi syndrome was recognised as being associated with the syndrome [2] and in 1965, a recessive X-linked pattern of inheritance was determined [3].

## Synonyms

Oculocerebrorenal syndrome, OCR

Lowe's oculocerebrorenal disease/syndrome, OCRL

Lowe's disease

Oculocerebrorenal dystrophy

## Epidemiology

Lowe's syndrome is a very rare disease, with estimated prevalence in the general population of approximately 1 in 500,000. According to the Lowe's Syndrome Association (LSA) in USA, the estimated prevalence is between 1 and 10 affected males in 1,000,000 inhabitants, with 190 living in the year 2000 (0.67 × million inhabitants). The Italian Association of Lowe's Syndrome (AISLO) estimated that there were 34 Lowe's syndrome patients (33 boys and one girl) living in Italy in the year 2005 (0.63 × million inhabitants). Lowe's Associations operate in several countries (USA, UK, France, Italy).

## Diagnostic criteria

Eye, central nervous system and kidney involvement are required for the diagnosis of Lowe's syndrome. Congenital bilateral cataract is present at the birth in all patients. Renal Fanconi syndrome may present in the first months of life and differ in severity between individuals. It may sometimes be asymptomatic or have an unusual clinical presentation. Involvement of the central nervous system is characterised by hypotonia and neonatal areflexia. Motor and mental developmental delays are present in infancy and stereotypic behaviour, such as temper tantrums and aggressiveness, is frequent during adolescence. Facial dysmorphisms are often present and consist in frontal bossing, deep-set eyes, chubby cheeks and fair complexion [4].

## Differential diagnosis

At birth, ocular involvement with bilateral cataract and hypotonia may be found in congenital infections (*i.e. *rubella), peroxisomal disorders, mitochondriopathies or myotonic congenital dystrophies or congenital myopathies (*i.e. *Muscle-Eye-Brain disease, MEB). The appearance of renal involvement excludes these alternative diagnoses within the first months of life.

## Clinical description

Signs and symptoms affecting the eye, central nervous system and the kidneys are characteristic of the syndrome.

### Eye

Dense cataract is present at birth in all patients. It develops *in utero *and is caused by altered migration of the crystalline embryonic epithelium [5]. Glaucoma (present in 50% of patients) with or without buphthalmos, is detected within the first year of life and sometimes even later. Sight sharpness is compromised by aphakia and, according to some authors, together with retinal dysfunction is responsible for nystagmus. Corneal and conjunctival cheloids (present in 25% of patients) further compromise the sight.

### Nervous system

A serious or very severe hypotonia is present at birth, often with absence of the deep tendon reflex. These manifestations may compromise suction and cause serious respiratory problems in the first period of life. Motor development is retarded and the autonomous gait becomes apparent generally after the third year. About 10% of patients show slight mental retardation. Mental retardation is moderate or severe, with an Intelligence Quotient (IQ) of 50 or less. Numerous patients (87%) show evidence of conduct disturbance with auto- and heteroaggressiveness, irritability, outbursts of anger and non-finalised behaviour. Obsessivecompulsive behaviour is typical [6]. Approximately 50% of the patients over 18 years old have seizures and up to 9% of the patients present febrile convulsions [7,8]. Cranial magnetic resonance imaging (MRI) may show a light ventriculomegaly and multiple periventricular cystic lesions in a bystander of the patients. They appear to stabilise with time and their clinical meaning has not yet been established [7,9]. Electromyogram (EMG) and motor and sensitive velocity are normal. No significant nerve and muscle pathologies are present and they are not useful for diagnosis. Habitual elevations in the levels of plasma creatine kinase (CPK) and transaminases are also found.

### Kidney

Renal disease is primarily characterised by renal Fanconi syndrome. The severity of the renal disease can vary significantly between patients and tends to worsen with age. At birth, many children are asymptomatic. The first symptoms generally develop during the first months of life and are generally related to renal bicarbonate, salt and water wasting, causing failure to thrive. Later, generally during the second decade of life, a significant number of patients develop chronic renal failure, which may lead to end-stage renal failure and requirement for dialysis. A minority of patients have been successfully treated with renal transplantation.

Symptoms related to the renal Fanconi syndrome include:

• low molecular weight proteinuria, which appears to be present in all patients and may be helpful for perinatal diagnosis;

• proximal renal tubular acidosis;

• renal phosphate wasting, leading to the development of renal rickets, osteomalacia and pathological fractures;

• hypercalciuria, leading to nephrocalcinosis and nephrolithiasis as a result of the Fanconi syndrome and of vitamin D therapy;

• aminoaciduria;

• hypokaliemia, mostly related to secondary hyperaldosteronism [10].

## Aetiology

The disease is caused by a reduction of phosphatidylinositol (4,5) bisphosphate 5 phosphatase, PtdIns (4,5)P2, activity below 10% in fibroblasts. Accumulation of phosphatidylinositol (4,5) bisphosphate (PiP2), the main substrate for this enzyme, in the Lowe cells and mutual disequilibrium of the phosphoinositides (that play a central role in cytoskeleton remodelling and in membrane traffic) cause the clinical picture at the birth and possibly the late complications. The following abnormalities have been reported in Lowe syndrome patients:

• altered cell signalling in the pathways that regulate endocytosis [11];

• defective actin cytoskeleton polymerisation, a process that is essential in the formation, maintenance and proper function of tight junctions and adherens junctions. These junctions have been demonstrated to be critical in renal proximal tubule function and in the differentiation of the lens [12];

• protein trafficking abnormalities.

While the genetic and enzymatic defects of Lowe syndrome have been thoroughly investigated, the mechanisms that lead to the severe clinical manifestations are still undefined (for review see [13]).

## Genetics

Lowe syndrome is transmitted by a X-linked mode of inheritance [3]. Two females who presented with the typical clinical picture associated with a balanced X-autosome translocation involving the Xq26 region [14,15], favoured the identification of the locus [16], and the gene was subsequently cloned by Nussbaum [17]. The causative gene *OCRL1 *contains 24 exons and encodes the OCRL1 protein, an inositol polyphosphate 5 phosphathase (belonging to the type II 5-phosphatase family), which localises to the trans-Golgi network [18]. To date, several mutations have been described: truncation mutations (nonsense, splicesite, frame-shift), missense mutations occurring in or outside the catalytic domain of *OCRL1 *and large deletions [19,20]. Somatic (*i.e. *mutations occurring in only some cell lines during foetal development) and germline mutations (*i.e. *mutations occurring in oocytes) have been identified in 4.5% of the patients [21] and should be considered in genetic counselling. As the disease is a recessive X-linked disorder, only males are usually affected but rare females with X-autosome translocations have been described. Recently, a female with a single 8 base-pair mutation was identified, with a normal karyotype and unfavourable lyonisation (Melis and Addis 2005, personal communication).

In patients with Dent's disease, a rare X-linked renal proximal tubulopathy without metabolic acidosis and neither ocular nor brain involvement, *OCRL1 *mutations (but lacked mutations in *CLCN5 *gene) were detected recently [22].

## Genetic counselling

*De novo *mutations are reported in 30% of affected males. Germline and somatic mosaicism has been identified in 4.5% of the patients. Lowe syndrome is a X-linked disease and the fathers are not carriers [21].

Mothers may be carriers with 25% possibility of having an affected boy, 25% possibility of having a carrier daughter, 25% possibility of having a unaffected boy, 25% possibility of having a non-carrier girl.

Mother may be a "non carrier" if the son presents a new mutation. In this case, chance of having another affected child is equal to that of the general population.

Mothers with a "germline mosaicism" can not be identified by slit-lamp, enzymatic and molecular testing.

All mothers of boys affected by Lowe's syndrome should undergo prenatal testing for possible germline mosaicism.

## Carrier detection

Female carriers of Lowe syndrome may be detected in 94% of cases by slit-lamp examination because of the presence of significantly punctuate white to grey opacities, distributed in a radial fashion in all layers of the lenticular cortex [23,24]. Using flanking linked markers [25], DNA analysis can reveal the same molecular defect (in a heterozygous state) as that previously identified in the patient. Since one third of the cases are due to new mutations, and germline mosaicism has been described, not all mothers of affected children show the mutation. Nevertheless, prenatal diagnosis should be offered to all affected families.

## Antenatal diagnosis

Enzymatic activity in cultured chorionic villi (at 9–11 weeks) or in cultured amniotic fluid cells (at 15–20 weeks) testing is available and should be performed if direct molecular testing is not possible, or when a germline mosaicism is suspected or when the pathogenic role of a detected mutation is questionable (*i.e. *missense mutation).

Genetic anomalies should be documented first in the proband. Direct molecular analysis can be performed on DNA extracted either from a chorionic villi sample or from cultured amniocytes. Screening for the *OCRL1 *gene mutation previously detected in the affected boy should be conducted. Linkage analysis can be done in informative families.

## Management including treatment

### Eye

Cataract should be removed early in order to avoid amblyopia. The early use of eye glasses or contact lenses improves visual function and consequently psycho-social skills. The ocular tone has to be tested frequently in order to diagnose glaucoma early and to treat it either with anti-glaucoma medication, or gonial or trabeculotomy surgery. Conjunctival or corneal cheloids are difficult to treat. Surgical lens implantation is not recommended; spectacles are to be preferred to contact lenses.

### Nervous system

Early targeted rehabilitation therapy is necessary to treat hypotonia and its complications. Tube feeding is not necessary in the early stages of the disease. An adequate psychological, pedagogical and occupational programme favours learning capacity and prevents frequent and serious behavioural crises during adolescence.

Areflexia is a peculiar state, which does not require treatment. Seizures require treatment with drugs specific for the symptoms. The behavioural problems occurring during adolescence and the obsessive-compulsive disorder require specific competence on the part of the health staff.

Drugs such as neuroleptics, stimulants, benzodiazepines, anti-depressives (tricyclic antidepressants and serotonin reuptake inhibitors) although adequately prescribed, are only partially efficacious. More promising results appear to be found with clomipramine, paroxetine and risperidone.

### Kidney

Renal tubular acidosis must be recognised and treated promptly with alkali supplements. These include citrates (sodium and/or potassium citrate) and sodium bicarbonate in variable doses and combinations, to maintain serum bicarbonate levels at around 20 mEq/l (doses may vary between 1–8 mEqKg/day, which should be divided into at least three separate doses).

Potassium citrate is particularly useful as it also helps to prevent nephrocalcinosis and tends to reduce renal calcium excretion.

If polyuria is present, patients should receive supplementary fluid. Sodium intake should be adjusted according to the extent of renal salt loss.

In infants and very young children, oral supplements should be promptly adjusted in case of diarrhoea. Intravenous infusions may be needed.

Rickets should be treated with oral phosphate supplements and vitamin D. Excessive amounts of vitamin D should be avoided as they may increase renal calcium excretion. Treatment should be targeted towards maintaining serum calcium and parathormone (PTH) levels within normal range and serum phosphate levels above 2–2.5 mg/dl.

Currently, there is no evidence that increasing the dietary protein content above normal recommendations is of benefit for these patients. Similarly, there is no evidence that Lcarnitine produces any improvement.

### Muscle and skeletal anomalies

Preventive treatments for the most common musculoskeletal complications are required to maintain articular mobility in order to avoid contractures. Osteopaenia and pathological fractures should be prevented by correct treatment of rickets. Standardised therapies (including the use of a corset and, if necessary, surgery) are required to prevent scoliosis.

### Other clinical signs

Cryptorchidism may improve with hormonal treatment and surgery is rarely required. Use of recombinant human growth hormone should be limited to patients with demonstrable growth hormone deficiency.

## Prognosis and quality of life

The longest reported survival is that of a 54 year-old patient. In the very first years of life, death may occur as a consequence of the renal disease or hypotonia, or increased susceptibility to infectious disease (respiratory or gastroenteric complications). The most frequent causes of death are: respiratory illness, epileptic seizures and sudden death, often while sleeping. Death usually occurs between the end of the second decade and the beginning of the fourth decade of life. The most remote cause of death is the renal tubulopathy, progressively evolving into renal insufficiency.

The quality of life depends on the extent of the mental and renal manifestations. Some patients may enjoy a discrete social life and assisted working activity.
